# An Audit of the Consistency and Quality of Letters From Choice Appointments in CAMHS South Edinburgh

**DOI:** 10.1192/bjo.2024.555

**Published:** 2024-08-01

**Authors:** Eileen Dempsey

**Affiliations:** NHS Lothian, Edinburgh, United Kingdom

## Abstract

**Aims:**

Hypothesis: Choice letters generated by Child & Adolescent Mental Health Services (CAMHS) South Edinburgh are not consistent in quality and content.

Aims:

1. To assess the consistency and quality of letters generated by clinicians following Choice appointments in CAMHS South.
2. To observe the range of resources shared in Choice letters.

**Methods:**

Patients were identified retrospectively from the team Choice diary.

Choice or Complex Choice appointments attended between 13^th^ October–27^th^ November 2023 at CAMHS South were included. See Soon appointments and appointments that were not attended or cancelled were excluded.

Standards to which all letters should adhere were devised from the CAPA (Choice & Partnership Approach) Book and the CAMHS South Choice Handbook.

Standards: current concerns, background information, patient goals, clinician impression, choices discussed, choices made, self-help agreed, services required, maximum two sides of A4, copy to patient, copy to referrer, sent within two weeks.

Letters were accessed via electronic records and analysed with a proforma.

A maximum of three letters per clinician was included.

A log of resources and frequency shared was kept.

**Results:**

57 appointments were attended, with 50 letters generated by 22 clinicians.

Adherence to standards in 50 available letters:

Current concerns 92%

Background information 96%

Patient goals 40%

Clinician impression 62%

Choices discussed 22%

Choices made 100%

Self-help agreed 52%

Services required 100%

Maximum two sides of A4 50%

Copy to patient 88%

Copy to referrer 100%

Sent within two weeks 72%

94 different resources were shared in the letters, with minimum 0 resources and maximum 19 resources per letter.

**Conclusion:**

There were areas of good quality and consistency in Choice letters, including documentation of current concerns, background information, and highlighting of required services. These are areas likely to feature in most assessments regardless of clinician background.

There were areas that require improvement, including documentation of goals, clinician impression, self-help agreed, and keeping to a maximum of two sides of A4. These areas are perhaps more obscure for different types of clinician.

A wide range of resources were shared in Choice letters with a considerable amount of variability in number of resources. This suggests differing levels of individualisation of resources to the patient.

Since this audit, CAMHS South have implemented additional Choice training, electronic canned text for letters, and collation of Choice guidance. There is a plan to re-audit following these interventions.

